# An assessment of the habitat preferences of European bison with airborne laser scanning data in forest ecosystem

**DOI:** 10.1038/s41598-023-45280-3

**Published:** 2023-10-20

**Authors:** Daniel Klich, Krzysztof Stereńczak, Maciej Lisiewicz, Maria Sobczuk, Angelika Nieszała, Wanda Olech

**Affiliations:** 1 Department of Animal Genetics and Conservation, Warsaw University of Life Sciences, Ciszewskiego 8, 02-786 Warsaw, Poland; 2 Department of Geomatics, Forest Research Institute, Sękocin Stary, 3 Braci Leśnej St., 05-090 Raszyn, Poland

**Keywords:** Behavioural ecology, Forest ecology

## Abstract

Research on habitat preferences is an important part of contemporary ecology. For the European bison, the classic approach to distinguishing habitat features is still being followed, but the limitations of this approach cannot provide the standard features of optimal habitats for this species. The study consisted in comparing analyses of the habitat preferences of European bison that were based on either classic forest typology (habitat types) or airborne laser scanning data. The data for these analyses were collected from telemetry collars on European bison in Białowieża Forest. The model based on airborne laser scanning features presented better parameters (percent of correctly classified cases and ROC) than the model based on habitat types. The results show that it is possible to find universal indicators of European bison’s preferences that are independent of local forest classification methodology. The indicators used suggest that European bison have a preference for forest habitats with low canopy cover and a small share of woody plants in the lower parts of the forest. Low canopy cover itself is not necessarily beneficial for European bison. Our study also indicates that airborne laser scanning is also useful in the assessment of habitat suitability for European bison in forest ecosystems.

## Introduction

Research on habitat preferences is an important part of contemporary ecology that arises from the need to understand the life demands of animals in a spatial context^1^. This is of particular importance for species management and conservation (e.g.,^2,3^) because proper recognition of optimal habitats helps in the effective conservation and reintroduction of a given species (e.g.,^4,5^). In the case of protected species, habitat quality assessment is of particular importance when launching reintroduction programs, which incur significant costs and effort. One such species is the European bison (*Bison bonasus* L.), for which conservation activities have been carried out since the restoration of the wild population^6^.

Classic methods (similar to those used for other large mammals) of assessing the presence of animals (direct observations, tracks, feces) were the basis of the first assessments of European bison’s habitat preferences. The habitat features that were chosen for analysis were mainly taken from forest maps, i.e., forest nomenclature was used. Borowski and Kossak^7^ found that about 75% of observations of European bison occurred in deciduous and mixed forests, but the study was performed without reference to available habitats. However, Krasiński^8^ indicated that European bison primarily preferred mixed forests, followed by deciduous forests, and then alder and coniferous forests. In subsequent studies, Krasiński et al.^9^ found that European bison visited deciduous forests (55%) more often than coniferous forests (42%) in the Polish part of Białowieża Forest; however, in the Belarusian part, 75% of observations occurred in coniferous forests. Daleszczyk et al.^10^, observed a change of habitat utilization by European bison over time: the use of coniferous forests decreased, while the use of deciduous forests and alderwoods increased. In mountains (Bieszczady), European bison preferred various habitat types, depending on the season. Wołoszyn-Gałęza et al.^11^ found that, in winter periods, European bison preferred deciduous and mixed forest areas located at lower altitudes above sea level; however, in the growing season these animals were much more likely to stay in coniferous forest areas with a low canopy cover. Other studies have indicated that European bison have a preference for coniferous stands^12,13^. Kuemmerle et al.^14^ showed that each European bison population’s preferences for particular types of habitats differed significantly. These results suggest that the standard features of optimal habitats for the European bison are still unknown. Kerley et al.^15^ indicates open habitats as most important for the European bison, but differences in individual locations are also expressed in European bison’s use of this type of habitats. In the lowlands, especially in large populations, the use of open areas was frequent^16,17^, but in the Bieszczady Mountains only about 10% of European bison locations were found outside of forest ecosystems^18^. European bison in western Poland used to show a strong preference for forest habitats, but this preference has changed towards open areas^19^. The classic division into forest habitat types was not able to explain these differences in preferences.

While progress has been made in collecting animal location data (from direct and indirect observations to telemetry), the classic approach to distinguishing habitat features (forest or Corine Land Cover (CLC) maps) is still being followed (e.g.,^14^). It is well known that forest typology (more precise than CLC) is not unambiguous, i.e., forest habitats with the same name may have different characteristics (e.g., crown cover, vertical structure of trees, etc.; [e.g.,^20^] and depend on the observer (e.g.,^21^). Moreover, forest typology differs between countries^22^, which means that its application at a larger spatial extent is not universal. Furthermore, forest habitat distributions are only updated at large intervals (in Poland every 10 years,^23^), which makes it difficult to find the key features of animals’ optimal habitats. These limitations make it difficult to go into greater detail when assessing habitat preferences. For this reason, forest ecosystems are usually divided into the classic forest habitat types that are still valid in Europe: coniferous, broadleaved and mixed forests^12,13,24^. Therefore, it seems that analyses of preferences should be based on the current characteristics of stands, not on classic forest habitat types. As indicated in numerous studies, this approach seems to be supported by stand characteristics that can be taken from classic forest habitat data: stand age^13,24,25^, dominant tree species^12,26^, but also light conditions and ground flora^24,26^. Also, the study conducted by Nieszała et al.^27^ indicated that the intensity of damage caused by European bison in forests was more dependent on stands’ canopy cover than on the dominant tree species and their age. Moreover, in the case of other ungulates (including typical grazers), the use of forest habitats has also found to be dependent on the physical characteristics of stands, such as canopy openness or abundance of ground flora (e.g.,^28–30^).

Currently, one technology with great potential for forest inventory and management applications is aircraft-mounted LiDAR (Light Detection and Ranging), known as Airborne Laser Scanning (ALS). A particular advantage of ALS in forestry applications is its capability to precisely characterize the three-dimensional (3D) structure of tree crowns. This three-dimensional structure is a fundamental physical element of habitat and has long been considered a key factor in biodiversity, especially in forests^31^. This information is potentially more valuable in forest applications than information from any other remote sensing techniques^32,33^. Structural features derived from ALS data can be used for ecological applications such as estimating forest resources and biodiversity. With the use of these data, it is possible to obtain information on tree species composition^34^, canopy gaps^35^, canopy cover^36^, stand structure^37^ or animals’ habitat preferences^38^, among others. As ALS provides detailed 3D data on vegetation structure and has the ability to calculate numerous metrics and model layers that are directly related to ecological aspects, it can be very useful in assessing habitats, organism-habitat relationships and biodiversity^39,40^. For example, in a study by Bässler et al.^41^ using ALS data, four Natura 2000 forest habitat classes were mapped on the basis of subtle differences in structure between habitat types. Bässler et al.’s^41^ study demonstrated that the use of ALS-derived structure variables can predict forest habitat types with accuracy similar to that of ground data on soil, vegetation composition and climate. Guo et al.^42^ conducted a cluster analysis on six ALS-derived habitat-related variables to classify vegetation structure into eight classes for forested areas. The authors concluded that the developed solution can be used as a base layer together with species and land cover data for forest resource planning, modelling species distribution and animal movements, and prioritizing conservation measures for critical habitat structures. Melin et al.^38^ used ALS data to investigate the role of forest structure in moose habitat use. The aim of their study was to investigate patterns of moose habitat selection during the year. In general, the authors found clear patterns and differences in habitat use between sexes and gained new and more accurate information about the role of forest structure for calving females. Their results show that data from ALS alone can provide valuable additional information about wildlife ecology. Studies that map the habitat preferences of animals are still scarce and have not yet been carried out on an animal as large as the European bison.

With this in mind, we sought to assess the habitat preferences of the European bison based on two modern methods of data acquisition: telemetry (for animal localization) and airborne laser scanning (to assess the quality of forest habitat features). In the study, we hypothesized that determining the current features of forest habitats using airborne laser scanning can explain the habitat preferences of the European bison better than the classic forest habitat types. Moreover, we hypothesize that the basic habitat features that explain the European bison’s preferences can be determined with airborne laser scanning.

## Methods

All experimental protocols (including immobilization of European bison) were approved by the Regional Directorate for Environmental Protection, based on the Act of 16 April 2004 on The Protection of Nature (Dz.U. 2004 nr 92 poz. 880). All methods were carried out in accordance with relevant guidelines and regulations.

The study consisted in comparing analyses of the habitat preferences of European bison that were performed using classic forest habitat types or airborne laser scanning data. These analyses were based on data collected from telemetry collars on European bison in Białowieża Forest, a forest complex that covers about 600 km^2^ in northeastern Poland. Białowieża Forest is a mixture of different habitat types, mainly coniferous, wet broadleaved forests, mesic rich broadleaved forest and successional stands^43^. This forest complex contains mature stands dominated by pedunculate (*Quercus robur* L.) and sessile (*Quercus petraea* (Matt.) Liebl.) oaks, common hornbeams (*Carpinus betulus* L.), small-leaved limes (*Tilia cordata* Mill.) with admixtures of Norway spruces (*Picea abies* (L.) H. Karst), Norway maples (*Acer platanoides* L.) and birches (*Betula pendula* Roth. and *Betula pubescens* Ehrh.)^44^. Białowieża Forest is inhabited by the largest (over 700 individuals) free-ranging population of European bison in Poland^45^.

The animals investigated in our study used mainly the forest ecosystem, but also open areas in the Białowieża forest complex. However, our study was related only to forest habitats for the following reasons. First, open ecosystems have a feeding function for European bison, and their choice depends on the quality of the food base. Other elements of vegetation structure appear to be important in the forest ecosystem and others in the open ecosystem, and they should be analyzed separately. The forest ecosystem provides European bison not only with food, but also with other life needs. Moreover, the quality of the food base in the forest floor depends largely on the vegetation in the upper parts of the forest. Therefore, assessing the structure of forest vegetation in the context of European bison preferences seems more meaningful, especially considering the fact that the establishment of new populations of this species in Central and Eastern Europe, where it mainly occurs, is based on large forest complexes.

### Telemetry data

For the purpose of this study, we used location data collected in 2014–2016 from the telemetry collars of 6 free-roaming European bison in Białowieża Forest. Collars were put on females, which generally show a schematic use of available habitats and a weak tendency to move, forcing them to search for optimal habitats at relatively short distances^6^. All females were adults and ranged in age from 6 to 17 years in 2015 (Table 1). The home range calculated by the MCP method, i.e. the area explored by collared European bison, ranged from 37.6 to almost 100 km^2^ (Table 1). The area used by European bison was much smaller, ranging from 16.8 to 42.7 km^2^, corresponding to between 17 and slightly more than 70% of the home range calculated by the MCP method. Telemetry collars were put on after prior pharmacological immobilization, performed with the consent of the Regional Director of Environmental Protection. These collars transmit a European bison’s location every hour. A total of 23,971 locations were used, including 14,391 in the growing season and 8880 in the winter season. Prior to analysis, the raw data was assessed in order to eliminate duplicate or erroneous records. For each individual, separately for the winter and the growing season, the home area was determined using the Kernel Density Estimation method (KDE95%). It was verified that the home ranges were within the study area (Białowieża Forest); if necessary, the borders were corrected to avoid observations for which habitat data was not present. Within each home range, the number of random points equaled the number of European bison locations (from the telemetry collars). All data points, both random points and from telemetry collars, were assigned to given forest habitat features. The forest habitat features originated, as indicated above, from forest maps and ALS.

**Table 1:** List of collared European bison females and their home range calculated by the MCP method and the Kernel Density Estimation method (95%).

Age in 2015 (years)	Home range with MCP (km^2^)	Home range with KDE95% (km^2^)
17	88.9	42.7
9	39.8	28.6
8	37.6	25.8
9	98.6	16.8
6	47.3	31.0
11	65.9	35.4

### Forest maps

We used public Digital Forest Maps (DFM) from four areas within Białowieża Forest: Białowieża National Park, Białowieża Forest District, Hajnówka Forest District and Browsk Forest District. These DFMs are implemented by the State Forests, which supports the management of forest resources. DFMs are useful in, among other, silvicultural planning, harvesting planning, analysis of management work, forest protection, hunting, or monitoring of changes that have occurred as a result of management work. They contain a wide range of stand information, of which we used forest habitat types in our work to investigate the habitat preferences of European bison. We have used forest maps to create a model that presents a reference to the results of the model based on data from ALS. For this reason, we chose a base model based on a general subdivision of forest habitats.

### Airborne laser scanning data and field measurements

The ALS dataset was acquired on 2–5 July 2015 using the LMS-Q680i full waveform system (Riegl, Horn, Austria). The acquired point cloud had an average density of 11 points/m^2^, the horizontal accuracy was ≤ 0.20 m, while the vertical accuracy was < 0.15 m. The flight altitude of 500 m resulted in a laser beam footprint of 0.25 m. To cover the entire study area, 135 individual flight lines were conducted. The ALS strips overlapped each other by 40% (field of view = 60°). The point cloud was acquired with a maximum scan angle of ± 30°. Spectral values from CIR (color-infrared) images were assigned to the ALS point cloud that were acquired at the same time. CIR aerial images were acquired using an UltraCam Eagle Camera with a 0.20 m ground sampling distance (GSD). In total, 1372 images were taken. Coverage between them was 90%/40% (forward/side overlap).

In order to interpret the results in detail and evaluate them visually, the study used 685 sample plots measured in the Białowieża Forest from July to October 2015. To conduct field measurements, the sample plots were distributed across the entire study area. The centers of individual plots were accurately measured using a real-time kinematic (RTK) receiver or a static geodetic class receiver for global navigation satellite systems. The position of each tree was calculated on the basis of the relationship between a specific tree and the center of the sample (distance and azimuth). During the field measurement, numerous tree-related characteristics were collected, such as tree species, tree height, diameter at breast height (DBH), crown length and tree viability. In addition, the visibility from above was determined for each tree.

### Extracting ALS variables

Using the ALS data, a set of metrics was calculated, from which five uncorrelated variables were selected for modelling. We selected metrics that make it possible to characterize the basic structural features of a forest, such as canopy height, crown cover, vertical structure, density of the lowest layers, and tree species composition (Table 2). All ALS statistics were calculated for a grid square with a cell size of 1ha (100 × 100 m). Due to the limited accuracy of the GPS position of the telemetry collars in the forest habitat, we used a cell of 1 ha. Location accuracy can drop significantly under tree canopy, especially on cloudy days. It can therefore be assumed that there may be a position error of over a dozen meters. Thus, 1 ha cells were used to reduce erroneous classification of the presence of animals in neighboring cells. Three variables were based directly on the vertical distribution of the point cloud: Hmax, Ground_to_all and CanRR. Hmax was determined by extracting the maximum tree height value from the ALS point cloud in each grid square. Ground_to_all was calculated as the ratio of points classified as ground to all return points in the ALS data. CanRR was calculated for all return points using the formula $\left( {{\text{avg}}\left( {\text{X}} \right) - {\text{min}}\left( {\text{X}} \right)} \right)/\left( {{\text{max}}\left( {\text{X}} \right) - {\text{min}}\left( {\text{X}} \right)} \right)$, where avg is average height of returns, min is minimal height of returns, and max is maximum height of returns. Canopy cover was counted as the ratio of grid coverage by the crown area of individual trees to the total grid area. Individual trees were extracted using the method described in Stereńczak et al.^46^. This method is based on finding local maxima and then extracting tree crowns by outlining minimum valleys on the Canopy Height Model with additional segmentation parameterization in three height ranges. The Broadleaved variable was calculated on the basis of the ratio between broadleaved trees to the total number of all tree species in the square grid. Tree species were classified according to the method described in Kamińska et al.^34^, which identified spruce, pine and broadleaved trees, also divided into alive and dead trees, with mean values for overall accuracy and Kappa of 94.3 and 0.93%, respectively. To achieve this, Kamińska et al.^34^ used ALS datasets (leaf-on and leaf-off) and a CIR dataset to color the point cloud from the leaf-on season. All data processing steps and the counting of metrics were performed in the R language with the following packages: *lidR*, *raster*, *rLiDAR*, *sf* and *sp*^47^.

**Table 2:** Features extracted from ALS data and derived products with definitions (Definition), ecological description (Description) and justification in the literature, indicating these features as important for the European bison (References).

Feature	Definition	Description	References
Hmax	Maximum height derived from the ALS point cloud	Describes the maximum height of a stand, i.e., indirectly reflecting stand age	^13,24,48^
Ground_to_all	Ratio of points classified as ground in relation to all points in the ALS point cloud	Characterizes the density and openness of a stand	^8,24,26,27^
CanRR	Canopy relief ratio calculated for all points from the ALS point cloud	Describes the vertical structure and stratification of a stand	^13,24,48^
Canopy cover	Percentage of area covered by crowns of individual trees	Characterizes the canopy closure of the trees in a stand	^8,24,26,27^
Broadleaved	Percentage of area covered by broadleaved trees in relation to all tree species	Describes the percentage of broadleaved trees and thus indirectly defines forest habitats	^8–10,14^

### Statistical analysis

The habitat preference was estimated with commonly used logistic regression models. In each model, the dependent variable was European bison presence data marked as 1, and random points marked as 0. In this approach, preference is considered as the response to the question of whether animals randomly occupy a certain habitat type (or a habitat with certain features). Thus, if European bison frequency in a given habitat is 0.5, the presence of the animals can be considered random; if the frequency is between 0 and 0.5, the animals are considered to avoid that habitat; and if the frequency is between 0.5 and 1, the habitat is considered preferred. Initially, binary mixed models were created in which an individual’s ID was the random factor in order to capture the repeatability of the measurements for an individual. Nevertheless, the mixed models showed fit uncertainties, therefore the random variable was ultimately dropped. In the first phase, four separate models were built: two models based on classic forest habitat types (one for winter and one for the vegetation period), and two models based on ALS (one for winter and one for the vegetation period). In the models based on classic forest habitat types, habitat was the explanatory variable (divided into three categories: coniferous, mixed, and broadleaved forests). In the models based on ALS data, a set of explanatory variables was used: Ground_to_all, CanRR, Hmax, canopy cover and Broadleaved, which was calculated based also on information from the CIR, simultaneously acquired and assigned to the ALS point cloud. To assess the significance of the variables in the model, we standardized all explanatory variables. We compared the models based on classic forest habitat types and airborne laser scanning separately for the winter and the vegetation periods using the percentage of correctly classified cases and the AUC (area under the curve). Then we built a new model for each of the three most important variables (Ground_to_all, Hmax and Broadleaved) for the winter and the vegetation periods; these models were based on raw values (not standardized) in order to compare the impact of these variables on the presence of European bison in these periods. We also created maps of preferred habitats based on airborne laser scanning data and logistic regression model output for raw data (for grid 1ha). We created two maps (one for the vegetation period and one for the winter period) for a binary value of potentially avoided habitats (probability of presence of European bison between 0 and 0.5), and potentially preferred habitats (probability of presence of European bison between 0.5 and 1). We also created two maps (one for the vegetation period and one for the winter period) with the potential preference of the European bison expressed as a continuous variable (from 0 to 1).

## Results

**Figure 1:**
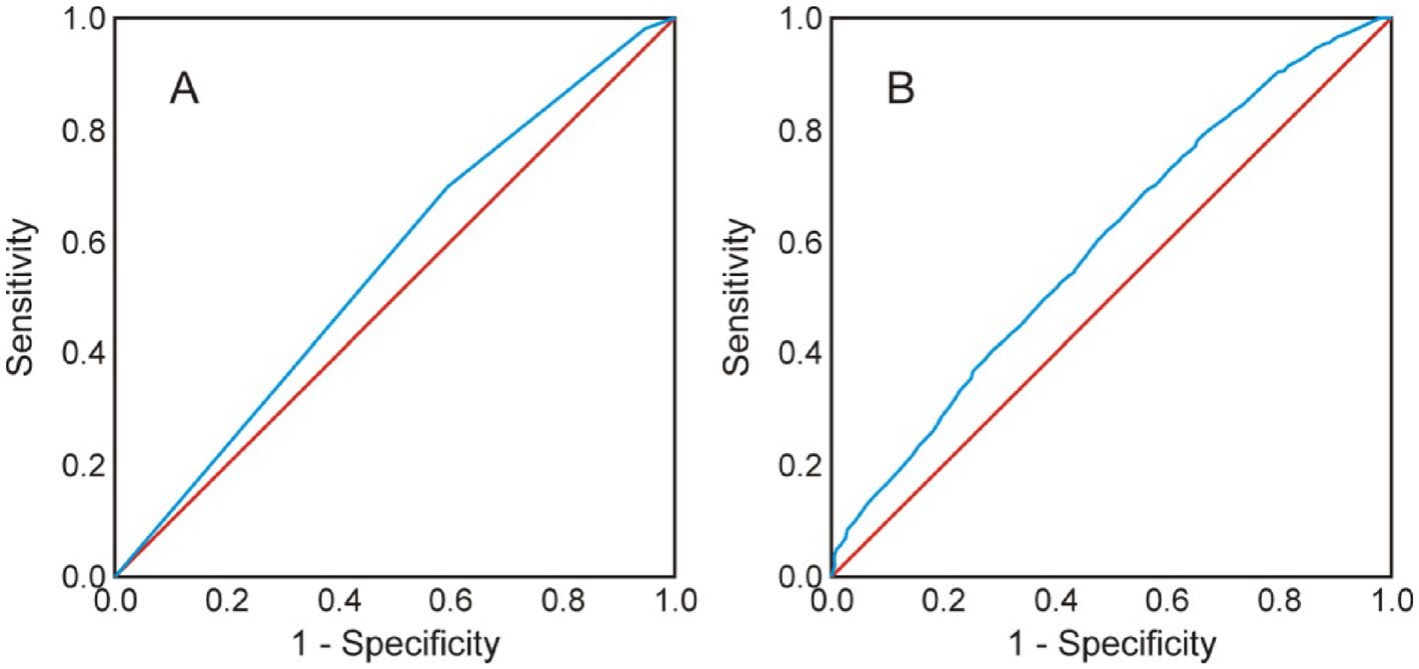
ROC curve of logistic regression models for the vegetation period with explanatory variables: (**A**) habitat types and (**B**) airborne laser scanning data. The red line represents no discrimination capacity of a given model to distinguish between positive and negative cases (AUC = 0.5). The blue line indicates the accuracy of a given model’s predictions.

**Figure 2:**
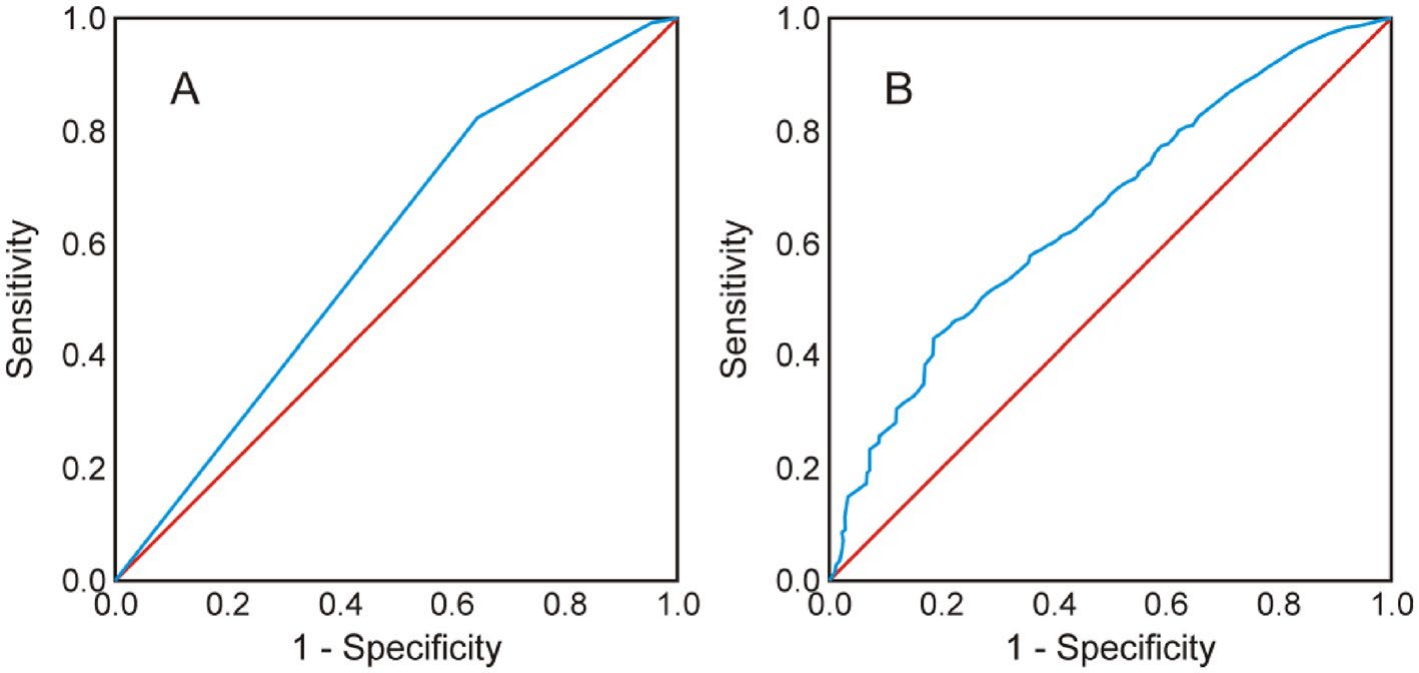
ROC curve of logistic regression models for the winter period with explanatory variables: (**A**) habitat types and (**B**) airborne laser scanning data. The red line represents a model’s lack of ability to distinguish between positive and negative cases (AUC = 0.5). The blue line indicates the accuracy of a given model’s predictions.

Both the classic forest habitat types and the habitat features obtained from airborne laser scanning significantly explained the habitat preferences of the European bison in the vegetation period (χ^2^ = 419.1, df = 2, *p* < 0.001 and χ^2^ = 1156.2, df = 5, *p* < 0.001 respectively). The model based on airborne laser scanning presented better parameters than the model based on classic forest habitat types, as expressed by the higher percentage of correctly classified cases (60.5 and 54.7% respectively) and the larger area under the curve (0.595 and 0.554 respectively, *p* < 0.001 in both cases) (Fig. 1). Differences were also observed between the models for the winter period, which also significantly explained the habitat preferences of the European bison (for the model based on classic forest habitat types, χ^2^ = 734.4, df = 2, *p* < 0.001; for the model based on airborne laser scanning, χ^2^ = 1159.2, df = 5, *p* < 0.001). For the model based on classic forest habitat types, 58.2% of cases were correctly classified; for the model based on airborne laser scanning, 60.5% of cases were correctly classified. The area under the curve was also larger for the ALS-based model (0.654) than for the model based on classic forest habitat types (0.591) (Fig. 2).

The effects of the models based on airborne laser scanning data differed between the vegetation and winter periods. For the vegetation period, canopy cover was not statistically significant and the effect size was small (Table 3), while in the winter period all variables significantly explained the presence of European bison in forest habitats (Table 4). In the vegetation period, the probability of the presence of European bison increased as the values of all variables in the model increased. Also, for the winter period, all variables showed a positive relation with the presence of European bison, except for canopy cover (Table 3). For the vegetation period, the most important variables in the model were Broadleaved (Exp(B) = 1.542), Ground_to_all (Exp(B) = 1.350) and Hmax (Exp(B) = 1.121). For the winter period, the same three variables were the most important in the model, but Hmax presented a higher impact on the European bison’s preference than Ground_to_all (EXP(B) = 1.468 and Exp(B) = 1.302, respectively).

**Table 3:** Effect of habitat features (standardized variables) acquired from airborne laser scanning on the habitat preferences of European bison in the logistic regression model for the vegetation period (for explanation of variables, see methods).

	B	SE	Wald	*P*	EXP (B)
Intercept	− 0.056	0.012	21.52	< 0.001	0.946
Ground_to_all	0.300	0.018	267.82	< 0.001	1.350
CanRR	0.056	0.013	17.81	< 0.001	1.058
Hmax	0.114	0.014	69.88	< 0.001	1.121
Canopy cover	− 0.022	0.015	2.26	0.133	0.978
Broadleaved	0.433	0.015	857.19	< 0.001	1.542

*B* beta coefficient of given predictor, *SE* standard error of beta coefficient, *Wald* Wald chi square test of the beta coefficient, *p* *p* value of the Wald chi square test, *EXP (B)* exponential value of the beta coefficient (odds ratio).

**Table 4:** Effect of habitat features (standardized variables) acquired from airborne laser scanning on the habitat preferences of European bison in the logistic regression model for the winter period (for explanation of variables, see methods).

	B	SE	Wald	*P*	EXP (B)
Intercept	− 0.078	0.016	22.98	< 0.001	0.925
Ground_to_all	0.264	0.025	110.14	< 0.001	1.302
CanRR	0.152	0.018	75.42	< 0.001	1.164
Hmax	0.348	0.018	434.55	< 0.001	1.468
Canopy cover	− 0.136	0.020	47.52	< 0.001	0.873
Broadleaved	0.446	0.021	443.66	< 0.001	1.562

*B* beta coefficient of given predictor, *SE* standard error of beta coefficient, *Wald* Wald chi square test of the beta coefficient, *p* *p* value of the Wald chi square test, *EXP (B)* exponential value of the beta coefficient (odds ratio).

**Figure 3:**
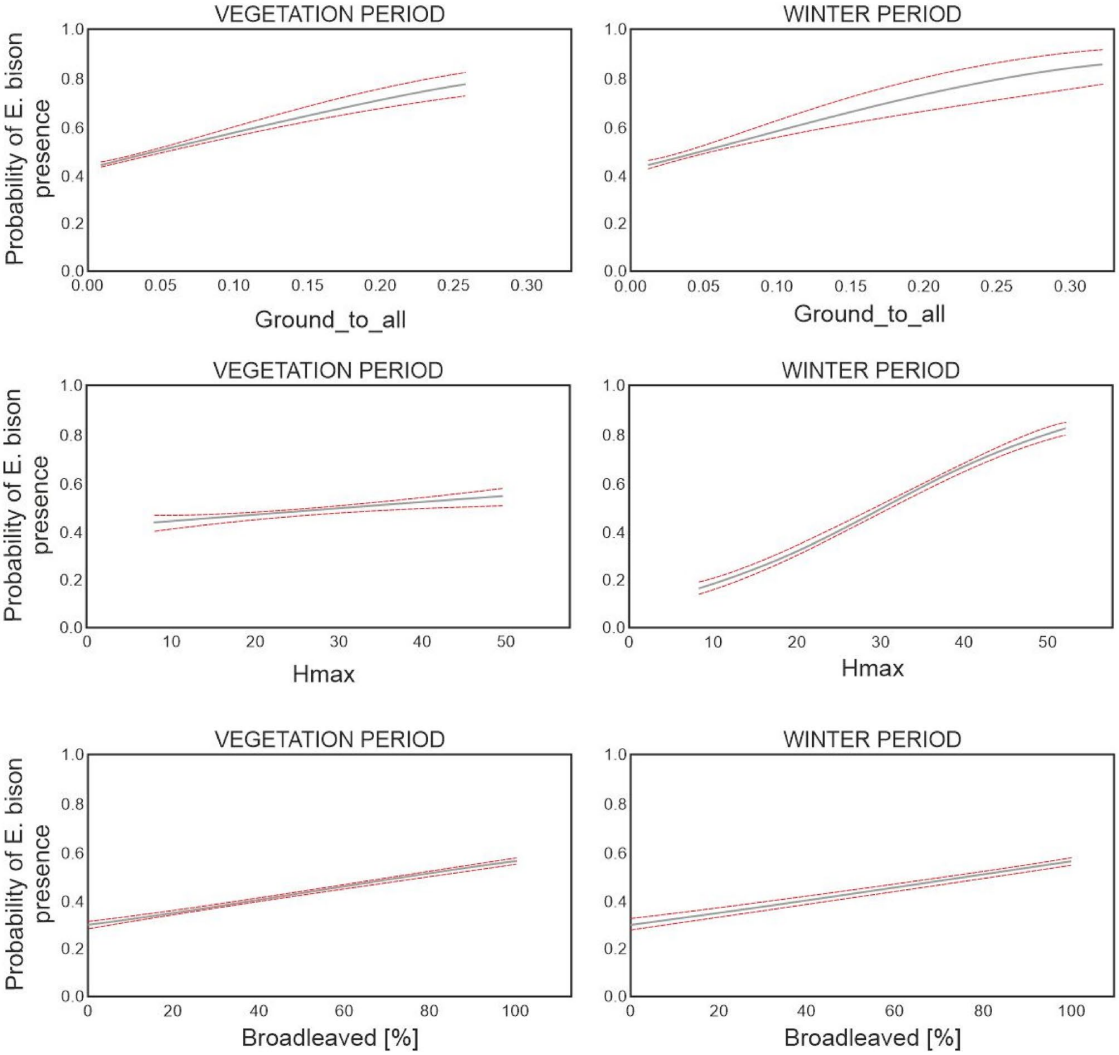
Probability of the presence of European bison in habitats with the Ground_to_all, Hmax and Broadleaved metrics for the vegetation and winter periods. The figure shows the increasing probability of European bison occurrence in forest habitats in both seasons with an increase of the Ground_to_all value and the percentage of Broadleaved species in the stand.

Based on models in which the three most important variables (Ground_to_all, Hmax and Broadleaved) were used as the only explanatory factor (regardless of all other variables), a distinct difference between the vegetation and winter periods can be observed (Fig.3). Ground_to_all presented similar trends for both periods, but European bison occupied habitats with higher values of this metric in the winter period (over 0.30) than in the vegetation period (0.25). The theoretical preference (where probability exceeds 0.5) was comparable to Ground_to_all in both periods (0.044 for the vegetation period and 0.045 for the winter period). European bison showed a preference for shorter tree stands in the winter period (H_max_ = 30 m) than in the vegetation period (H_max_ = 34 m). Nevertheless, the increased probability of the presence of European bison was more pronounced with an increase of the maximum tree height in the winter period. The proportion of broadleaved trees in the habitat presented a similar trend in both seasons. European bison preferred stands with a proportion of broadleaved trees of at least 75% (for the vegetation period) or 78% (for the winter period).

**Figure 4:**
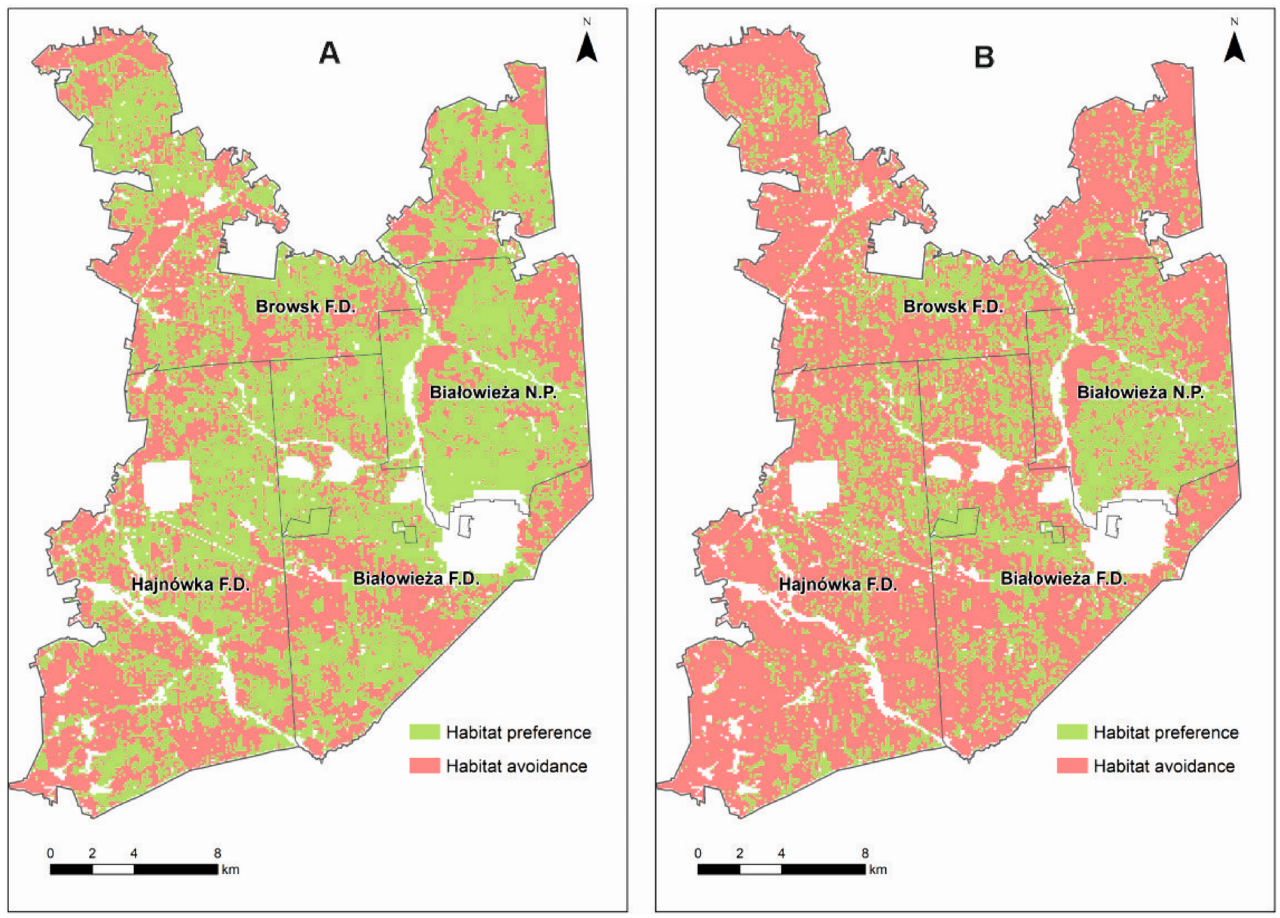
Map showing the predicted preference of European bison for forest habitats in the Polish part of Białowieża Forest on the basis of ALS metrics (binary output) in (**A**) the vegetation and (**B**) the winter period. The map was generated in ArcMap Software, ver. 10.3 (https://desktop.arcgis.com/en/arcmap/).

The binary output map (Fig.4) shows that habitat features preferred during the vegetation period are more common in Białowieża Forest than habitat features preferred during the winter period. This is reflected in the total area of the preferred forest habitats (calculated probability above 0.5), which is almost twice as large in the vegetation period than in the winter period (31,016 and 16,719 ha, respectively). During the growing season, European bison show a preference not only for the entire central part of Białowieża Forest (including almost the entire area of Białowieża National Park), but also large areas in the south and north of this forest complex. In the winter period, the preferred habitats are also in the central part of Białowieża Forest, including the western part of Białowieża National Park, the northern part of Białowieża Forest District, the eastern part of Hajnówka Forest District, and the southern part of Browsk Forest District. Other large areas of preferred habitats are the northeastern and northwestern part of Browsk Forest District and the southern part of Białowieża Forest District.

**Figure 5:**
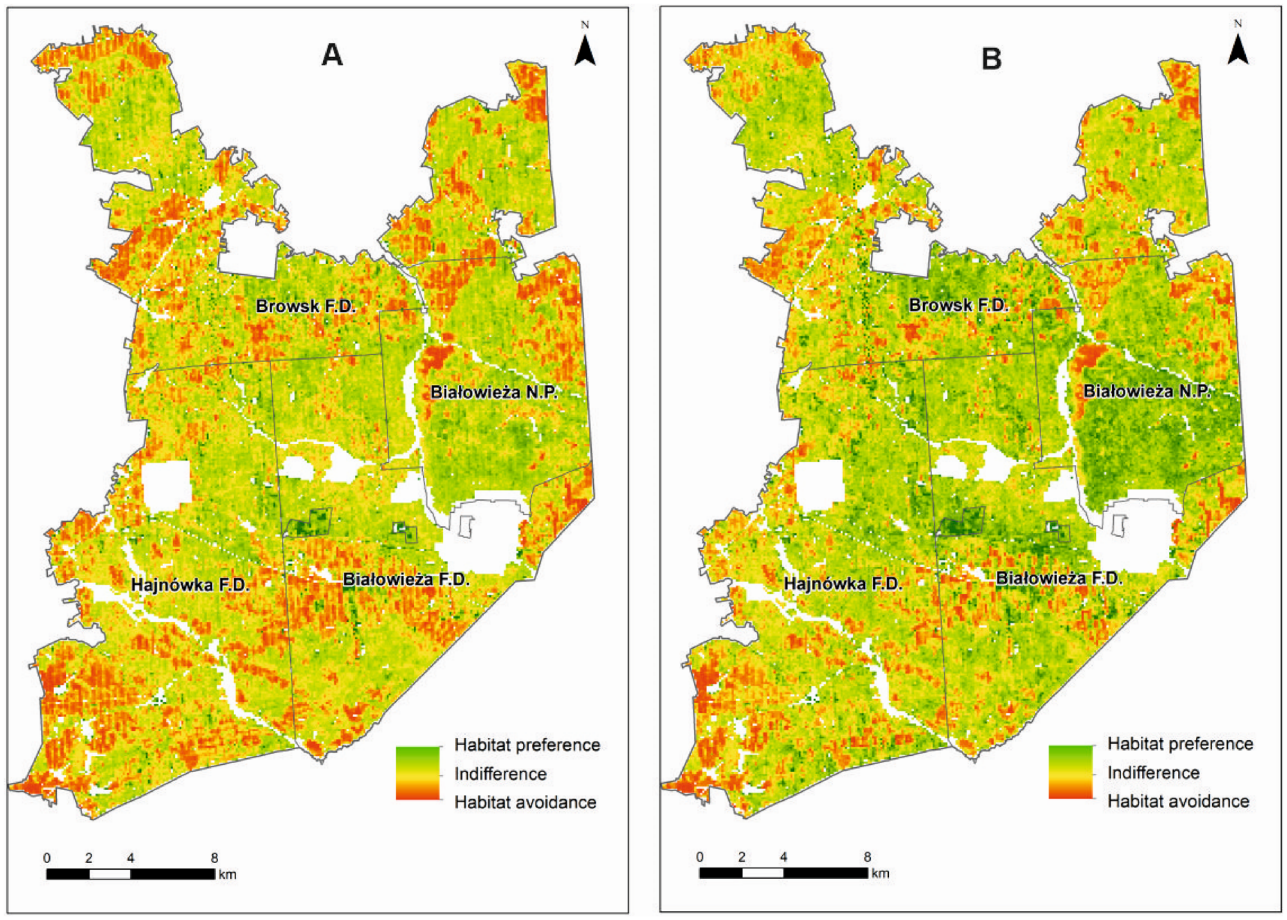
Map presenting the predicted preference of European bison for forest habitats in the Polish part of Białowieża Forest on the basis of ALS metrics (continuous output) in (**A**) the vegetation and (**B**) the winter period. The map was generated in ArcMap Software, ver. 10.3 (https://desktop.arcgis.com/en/arcmap/).

The map with a continuous output (Fig.5) shows the areas avoided by European bison. Moreover, this map shows a more pronounced preference and greater tolerance of suboptimal habitats in the winter period than in the vegetation period. The greater tolerance of suboptimal habitats is expressed in a smaller area of mostly avoided habitats on the map. However, the distribution of mostly avoided habitats is generally similar in the vegetation and the winter periods.

## Discussion

According to our hypothesis, airborne laser scanning could explain the habitat preferences of European bison better than the classic forest habitat types (higher percentage of correctly classified cases and larger ROC), but the performance of the ALS data was not markedly better. Nevertheless, the conducted analysis allowed us to indicate the basic habitat features that explain European bison’s preferences. Therefore, our results confirm the possibility of using ALS for a large mammal with unclear preferences for forest habitats, i.e., European bison. Similarly, ALS has been successfully used to assess habitat suitability for other species^38,49–51^ and to assess the effects of habitat quality on their distribution or behavior^39,52^. Nevertheless, some of the results in this study were surprising, thus requiring careful analysis of this phenomenon in relation to current knowledge.

The proportion of broadleaved trees was the most important habitat feature that explained the presence of European bison in forest habitats, during both the vegetation and the winter periods. It can be assumed that this result reflects the most frequently reported preference of European bison for broadleaved forest habitats, as has been demonstrated in previous studies (e.g.,^9,14,53,54^). However, attention should be paid not only to the indicated preference of the European bison for old stands with a multistorey structure^13,24,48,54^, but also to forest habitats with old spruce in the upper layer with a loose canopy and deciduous trees in lower layers (e.g.,^8^). Such stands are classified as mixed forest and are often present in Białowieża Forest^55^. In recent years, as a result of dead spruce, deciduous species develop more often in the second layer of old spruce stands^56,57^. Therefore, a high percentage of broadleaved trees does not always mean that broadleaved trees predominate in the uppermost stand layer. This is because the percentage of the “broadleaved” measure represents the total area of leaf types (coniferous or broadleaved) viewed from above^46^. The crowns of mature broadleaved trees usually spread more than the conical crowns of conifers^34^. This indicates that the dominance of broadleaved species can also be caused by trees in the lower stand layers. In addition, the “Broadleaved” measure indicates the area of tree canopy covered by a tree species, which differs from the classic forest stand typology, where indicator plants define the stand type^23^.

The above assumptions are consistent with the second important stand characteristic, namely Hmax. It should be noted that in winter Hmax reflects European bison’s preferences very well when it is the only variable in the model, and the preference for habitats with an Hmax value of 50 m is extremely strong, reaching the value of 0.8. This level of preference is comparable to open areas in lowlands (e.g.,^25,58^). Therefore, it can be assumed that not the habitat type itself but characteristics such as stand height (old stands) and an abundance of deciduous trees are of great importance for European bison’s habitat selection. It should be noted that the Hmax value itself is of less importance as the maximum value of the height of the stands in which the European bison occurred. This is due to the fact that bison visited habitats with a higher Hmax value during the growing season than in winter, which could also be an effect of other correlated parameters (age, stand density, etc.).

Surprisingly, canopy cover was of lesser importance in evaluating European bison’s preferences and was not statistically significant in the vegetation period. In contrast, Ground_to_all was the proxy for forest habitat features that played a much larger role in the models. This result is even more surprising because in previous studies an open canopy was often, but not always, indicated as a characteristic of stands preferred by European bison (e.g.,^16,26,54^). It should be remembered that these two characteristics (canopy cover and Ground_to_all) do not mean the same thing. Ground_to_all is the percentage of points that reach the ground and does not correlate with canopy cover, since the former variable describes forest vertical structure, while the latter one describes the horizontal structure of the top tree layer. This means that “ground to all” also indirectly indicates the presence of an understory in the habitat as the emitted laser pulse is partially absorbed, thus not all the signal is returned. An understory often develops when canopy closure is low (e.g.,^59,60^), which inhibits the growth of ground flora (e.g.,^61,62^), especially herbs and grasses, which are the main diet of European bison^7,63,64^. CanRR also seems to confirm this interpretation, as this indicator tells us what percentage of laser points were in the upper part of the tree canopies. Both indicators suggest European bison’s preference for forest habitats with low canopy cover with a small share of woody plants in the lower parts of the forest. Low canopy cover itself is not necessarily beneficial for European bison. This is ecologically justified, because open canopy can be associated with dense undergrowth that restricts the development of ground flora, which provides the bison’s nutritional needs. For this reason, Ground_to_all, supported by CanRR, appears to be an important feature of forest habitats with an adequate food supply for European bison.

The search for standard features is justified in the context of European bison conservation on a continental scale. This is of particular importance in the context of the ongoing need for active measures to protect European bison, including reintroduction^65^. The above results show that it is possible to find indicators of European bison’s preferences that are independent of local forest type classification methodology. However, the models with ALS were not remarkably better than the models based on classic forest typology. This may be due to the European bison’s unclear preferences and habitat plasticity (e.g.,^14,64,66^). The reason for the slightly better ALS-based models could also be the settings used in our work. The cell size of 1 ha made it possible to minimize the erroneous location of European bison in given cell, but a smaller grid would allow for a more accurate description of forest habitats. The use of more accurate locations would probably allow for better model indices. The metrics we used also show only the basic features of forest habitats that determine the selection of particular habitats by European bison. The metrics used in Melin et al.’s^38^ study described the proportion of echoes above five meters (p_canopy), echoes that hit the ground (p_ground) and echoes between the ground and a height of five meters (p_shrub).

Our study indicates, however, that secondary metrics such as Can_RR or Ground_to_all may also be helpful in habitat preference assessment. In addition, the Broadleaved and canopy cover variables that we used help to reflect the actual structure of the forest more accurately and thus also habitat preferences. For this reason, it is necessary to conduct further studies, including analyses in forest complexes with a domination of coniferous stands. What is more, it is worth considering looking for other ALS-based indicators which could better characterize the habitat preferences of European bison. Many other habitat features have been demonstrated that may explain the use of certain habitats by European bison^16,26,67^, and the effects of these features vary locally^14,54^. In addition, it is possible that a combination of habitat and ALS data would provide a better understanding of how European bison use forest habitats. However, it was not the aim of this study to show the full range of factors explaining the preferences of European bison. Nevertheless, it has been shown that the data from ALS can be used to analyze the preferences of European bison. This is important because ALS is intensively used worldwide and its availability is increasing (e.g.,^68^). The maps produced in our study showing the predicted preference of European bison demonstrate the possibility of a global assessment of habitat suitability of large areas, including those that are difficult to access. This creates new opportunities for active species conservation.

## Data Availability

The datasets generated during the current study are available from the corresponding author on reasonable request.

## References

[1] Levin SA (1992). The problem of pattern and scale in ecology. Ecology.

[2] Canadas A, Sagarminaga R, De Stephanis R, Urquiola E, Hammond PS (2005). Habitat preference modelling as a conservation tool: Proposals for marine protected areas for cetaceans in southern Spanish waters. Aquat. Conserv. Mar. Freshw. Ecosyst..

[3] Stamps JA, Swaisgood RR (2007). Someplace like home: Experience, habitat selection and conservation biology. Appl. Anim. Behav. Sci..

[4] Owen-Smith N (2003). Foraging behavior, habitat suitability, and translocation success, with special reference to large mammalian herbivores. Anim. Behav. Wildl. Conserv..

[5] Morrison ML, Mathewson HA (2015). Wildlife Habitat Conservation: Concepts, Challenges, and Solutions.

[6] Olech, W. & Perzanowski K. European Bison (*Bison bonasus*) Strategic Species Status Review 2020. IUCN SSC Bison Specialist Group and European Bison Conservation Center, Warsaw 1–138 (2022).

[7] Borowski S, Kossak S (1972). The natural food preferences of the European bison in seasons free of snow cover. Acta Theriol..

[8] Krasiński ZA (1978). Dynamic and structure of the European bison population in the Białowieża Primeval Forest. Acta Theriol..

[9] Krasiński ZA, Krasińska M, Bunevich A (1999). Free-ranging population of lowland European bison in the Białowieża Forest. Parki Narodowe Rezerw. Przyr..

[10] Daleszczyk K, Krasińska M, Krasiński ZA, Bunevich AN (2007). Habitat structure, climatic factors, and habitat use by European bison (*Bison bonasus*) in Polish and Belarusian parts of the Białowieża Forest, Poland. Can. J. Zool..

[11] Wołoszyn-Gałęza A, Perzanowski K, Januszczak M, Pagacz S (2016). Habitat preferences of a European bison (*Bison bonasus*) populaton in the Carpathian Mountains. Ann. Zool. Fennici.

[12] Hass Brandtberg N, Dabelsteen T (2013). Habitat selection of two European bison (*Bison bonasus*) on the Danish Island Bornholm. Eur. Bison Conserv. Newsl..

[13] Červený J (2014). Daily activity rhythm and habitat use of the semi-free European bison herd during the growing season. Lesn. Čas. For. J..

[14] Kuemmerle T (2018). One size does not fit all: European bison habitat selection across herds and spatial scales. Landsc. Ecol..

[15] Kerley GI, Kowalczyk R, Cromsigt JP (2012). Conservation implications of the refugee species concept and the European bison: King of the forest or refugee in a marginal habitat?. Ecography.

[16] Kowalczyk R (2013). Movements of European bison (*Bison bonasus*) beyond the Białowieża Forest (NE Poland): Range expansion or partial migrations?. Acta Theriol..

[17] Sobczuk M, Olech W (2016). Damage to the crops inflicted by European bison living in the Knyszyn Forest. Eur. Bison Conserv. Newsl..

[18] Perzanowski K, Januszczak M, Wołoszyn-Gałęza A, Klich D (2021). Wieloletnia dynamika areału populacji żubrów *Bison bonasus*, na terenie nadleśnictw bieszczadzkich. Sylwan.

[19] Grzegorzek, M. Telemetria GPS jako element czynnej ochrony żubrów w województwie zachodniopomorskim. Ing Thesis, Uniwersytet Technologiczno-Przyrodniczy w Bydgoszczy (2012).

[20] F.A.O. (Food and Agriculture Organization). Global Forest Resources assessment. (United Nations, Rome, 2015).

[21] Roper BB, Scarnecchia DL (1995). Observer variability in classifying habitat types in stream surveys. N. Am. J. Fish. Manag..

[22] Barbati A, Corona P, Marchetti M (2007). A forest typology for monitoring sustainable forest management: The case of European forest types. Plant Biosyst..

[23] Talarczyk A (2014). National forest inventory in Poland. Balt. For..

[24] Cătănoiu S, Deju R (2008). Monitoring of the European Bison—*Bison bonasus* L.—using radio telemetry within the aclimatisation enclosure, in the Vanatori Neamt Nature Park. Studia Universitatis “Vasile Goldis”. Seria Stiint. Vietii.

[25] Marozas V, Kibiša A, Brazaitis G, Jõgiste K, Šimkevicius K, Bartkevicius E (2019). Distribution and habitat selection of free-ranging European Bison (*Bison bonasus* L.) in a Mosaic Landscape—a lithuanian case. Forests.

[26] Charytanowicz M, Perzanowski K, Januszczak M, Wołoszyn-Gałęza A, Kulczycki P (2022). Habitat suitability for wisents in the Carpathians–a model based on presence only data. Ecol. Inform..

[27] Nieszała A, Klich D, Perzanowski K, Januszczak M, Wołoszyn-Gałęza A, Olech W (2022). Debarking intensity of European bison in the Bieszczady Mountains in relation to forest habitat features. For. Ecol. Manag..

[28] Kuijper DP, Cromsigt JP, Churski M, Adam B, Jędrzejewska B, Jędrzejewski W (2009). Do ungulates preferentially feed in forest gaps in European temperate forest?. For. Ecol. Manag..

[29] Massé A, Côté SD (2012). Linking habitat heterogeneity to space use by large herbivores at multiple scales: From habitat mosaics to forest canopy openings. For. Ecol. Manag..

[30] Klich D, Grudzień M (2013). Selective use of forest habitat by Biłgoraj horses. Belg. J. Zool..

[31] MacArthur RH, MacArthur J (1961). On bird species diversity. Ecology.

[32] Lefsky MA, Cohen WB, Spies TA (2001). An evaluation of alternate remote sensing products for forest inventory, monitoring, and mapping of Douglas-fir forests in western Oregon. Can. J. For. Res..

[33] Maltamo M, Malinen J, Packalén P, Suvanto A, Kangas J (2006). Nonparametric estimation of stem volume using airborne laser scanning, aerial photography, and stand-register data. Can. J. For. Res..

[34] Kamińska A, Lisiewicz M, Stereńczak K, Kraszewski B, Sadkowski R (2018). Species-related single dead tree detection using multi-temporal ALS data and CIR imagery. Remote Sens. Environ..

[35] Dobrowolska D, Piasecka Ż, Kuberski Ł, Stereńczak K (2022). Canopy gap characteristics and regeneration patterns in the Białowieża forest based on remote sensing data and field measurements. For. Ecol. Manag..

[36] Arumäe T, Lang M (2018). Estimation of canopy cover in dense mixed-species forests using airborne lidar data. Eur. J. Remote Sens..

[37] Bottalico F (2017). Modeling Mediterranean forest structure using airborne laser scanning data. Int. J. Appl. Earth Obs. Geoinform..

[38] Melin M, Matala J, Mehtätalo L, Pusenius J, Packalen P (2016). Ecological dimensions of airborne laser scanning—Analyzing the role of forest structure in moose habitat use within a year. Remote Sens. Environ..

[39] Hill R, Hinsley SA, Broughton RK, Maltamo M (2014). Assessing habitats and organism–habitat relationships by airborne laser scanning. Forestry Applications of Airborne Laser Scanning.

[40] Müller J, Vierling K, Maltamo M (2014). Assessing biodiversity by airborne laser scanning. Forestry Applications of Airborne Laser Scanning.

[41] Bässler C, Stadler J, Müller J, Forster B, Gottlein A, Brandl R (2011). LiDAR as a rapid tool to predict forest habitat types in Natura 2000 networks. Biodivers. Conserv..

[42] Guo X, Coops NC, Tompalski P, Nielsen SE, Bater CW, John Stadt J (2017). Regional mapping of vegetation structure for biodiversity monitoring using airborne lidar data. Ecol. Inform..

[43] Sokołowski AW (2004). Lasy Puszczy Białowieskiej.

[44] Faliński JB (1986). Vegetation dynamics in temperete forests. Ecological Studies in Białowieża Forest.

[45] Raczyński J (2022). The European Bison Pedigree Book 2021.

[46] Stereńczak K, Kraszewski B, Mielcarek M, Piasecka Ż, Lisiewicz M, Heurich M (2020). Mapping individual trees with airborne laser scanning data in an European lowland forest using a self-calibration algorithm. Int. J. Appl. Earth Obs. Geoinform..

[47] R Core Tea. *R: A language and environment for statistical computing*. R Foundation for Statistical Computing, Vienna, Austria (2022). https://www.R-project.org/. Accessed 20 Nov 2022.

[48] Krasińska M, Krasiński Z (2013). European Bison: The Nature Monograph.

[49] Melin M, Packalén P, Matala J, Mehtätalo L, Pusenius J (2013). Assessing and modeling moose (*Alces alces*) habitats with airborne laser scanning data. Int. J. Appl. Earth Obs. Geoinform..

[50] Mononen L (2018). Usability of citizen science observations together with airborne laser scanning data in determining the habitat preferences of forest birds. For. Ecol. Manag..

[51] de Vries JPR, Koma Z, WallisDeVries MF, Kissling WD (2021). Identifying fine-scale habitat preferences of threatened butterflies using airborne laser scanning. Divers Distrib..

[52] Boelman N (2016). Airborne laser scanning and spectral remote sensing give a bird’s eye perspective on arctic tundra breeding habitat at multiple spatial scales. Remote Sens. Environ..

[53] Kuemmerle T (2011). Predicting potential European bison habitat across its former range. Ecol. Appl..

[54] Smagol V (2022). characteristics of European bison (*Bison bonasus*) in Ukraine. Eur. J. Wildl. Res..

[55] Jaroszewicz B, Cholewińska O, Gutowski JM, Samojlik T, Zimny M, Latałowa M (2019). Białowieża forest—A relic of the high naturalness of European forests. Forests.

[56] Brzeziecki B (2018). Problem of a massive dying-off of Norway spruce stands in the 'Białowieża Forest' Forest Promotional Complex. Sylwan.

[57] Miścicki S, Kuberski Ł, Paluch R, Pilch K, Stereńczak K, Stereńczak K (2022). Wood resources of the Białowieża Forest in 2015–2019—status and dynamics. The Current State of Białowieża Forest Based on the Results of the LIFE+ ForBioSensing Project.

[58] Zikmund M, Ježek M, Silovský V, Červený J (2021). Habitat selection of semi-free ranging European bison: Do bison preferred natural open habitats?. Cent. Eur. For. J..

[59] Brosofske KD, Chen J, Crow TR (2001). Understory vegetation and site factors: Implications for a managed Wisconsin landscape. For. Ecol. Manag..

[60] Majasalmi T, Rautiainen M (2020). The impact of tree canopy structure on understory variation in a boreal forest. For. Ecol. Manag..

[61] Conway DW, Parker AJ, Parker KC (1997). Understory light regime, shrub layer and sand pine (*Pinus clausa*) regeneration in four scrub stands. Am. Midl. Nat..

[62] Messier C, Parent S, Bergeron Y (1998). Effects of overstory and understory vegetation on the understory light environment in mixed boreal forests. J. Veg. Sci..

[63] Zielke L, Wrage-Mönnig N, Müller J (2017). Seasonal preferences in diet selection of semi-free ranging European bison (*Bison bonasus*). Eur. Bison Conserv. Newsl..

[64] Kowalczyk R (2019). Foraging plasticity allows a large herbivore to persist in a sheltering forest habitat: DNA metabarcoding diet analysis of the European bison. For. Ecol. Manag..

[65] Olech W, Klich D, Perzanowski K (2019). Development of a new action plan for the European bison. Oryx.

[66] Łopucki R (2023). Individual differentiation of habitat preferences indicate high flexibility in habitat use by European bison (*Bison bonasus*). Glob. Ecol. Conserv..

[67] Kuemmerle T (2010). European bison habitat in the Carpathian Mountains. Biol. Conserv..

[68] Moudrý V (2023). Vegetation structure derived from airborne laser scanning to assess species distribution and habitat suitability: The way forward. Divers. Distrib..

